# Low intensity interventions for Obsessive-Compulsive Disorder (OCD): a qualitative study of mental health practitioner experiences

**DOI:** 10.1186/s12888-017-1238-x

**Published:** 2017-02-22

**Authors:** Judith Gellatly, Rebecca Pedley, Christine Molloy, Jennifer Butler, Karina Lovell, Penny Bee

**Affiliations:** 1 Division of Nursing, School of Health Sciences, Faculty of Biology, Medicine and Health, The University of Manchester, Manchester Academic Health Science Centre, Manchester, M13 9PL UK; 2 Division of Psychology and Mental Health, Faculty of Biology, Medicine and Health, University of Manchester, Manchester Academic Health Science Centre, Manchester, M13 9PL UK

**Keywords:** Acceptability, Computerised cognitive behaviour therapy, Guided self-help, Low intensity intervention obsessive-compulsive disorder, Qualitative

## Abstract

**Background:**

Obsessive-compulsive disorder (OCD) is a debilitating mental health disorder that can substantially impact upon quality of life and everyday functioning. Guidelines recommend pharmacological and psychological treatments, using a cognitive behaviour therapy approach (CBT) including exposure and response prevention, but access has generally been poor. Low intensity psychological interventions have been advocated. The evidence base for these interventions is emerging but there is a paucity of information regarding practitioners’ perceptions and experiences of supporting individuals with OCD using this approach.

**Methods:**

Qualitative interviews were undertaken with psychological wellbeing practitioners (PWPs) (*n* = 20) delivering low intensity psychological interventions for adults with OCD within the context of a large pragmatic effectiveness trial. Interviews explored the feasibility and acceptability of delivering two interventions; guided self-help and supported computerised cognitive behaviour therapy (cCBT), within Improving Access to Psychological Therapies (IAPT) services in NHS Trusts. Interviews were recorded with consent, transcribed and analysed using thematic analysis.

**Results:**

PWPs acknowledged the benefits of low intensity psychological interventions for individuals experiencing OCD symptoms on an individual and population level. Offering low intensity support provided was perceived to have the opportunity to overcome existing service barriers to access treatment, improve patient choice and flexibility. Professional and service relevant issues were also recognised including self-beliefs about supporting people with OCD and personal training needs. Challenges to implementation were recognised in relation to practitioner resistance and intervention delivery technical complications.

**Conclusions:**

This study has provided insight into the implementation of new low intensity approaches to the management of OCD within existing mental health services. Benefits from a practitioner, service and patient perspective are identified and potential challenges highlighted.

**Trial registration:**

Current Controlled Trials: ISRCTN73535163. Date of registration: 5 April 2011.

## Background

Obsessive compulsive disorder (OCD) is a debilitating and distressing mental health problem. It is characterised by the presence of obsessions; uncontrollable distressing repetitive thoughts, impulses or images and compulsions; ritualised behaviours or mental acts that are carried out with the aim to reduce distress. OCD negatively affects an individual’s quality of life and significantly interferes with everyday functioning and activities [1]. The impact of OCD can also extend to the lives of friends and families and the wider society [2]. If not treated improvement in symptoms is unlikely [3, 4]. The World Health Organisation (WHO) considers it to be the 11th leading cause of health burden [5] and has a lifetime prevalence of 2-3% [5].

Similar to the management of other mental health problems, such as anxiety and depression, access to recommended treatments within the UK mental health system has in the past failed to meet demand with many people faced with long waiting times [6, 7]. Whilst treatment guidelines recommend both pharmacological and psychological treatments, research has highlighted people prefer psychological therapies to medication [8]. Despite the existence of effective and acceptable treatments, many patients are faced with a variety of access barriers including lack of trained practitioners, stigma and costs of treatment [9–14]. Ensuring timely access to appropriate psychological therapies has therefore become the focus of recent developments [15]. In the UK psychological therapy is delivered via the IAPT programme [16] that was implemented to optimise access to effective treatments for people experiencing depression and anxiety disorders.

UK National Institute of Health and Care Excellence (NICE) OCD and body dysmorphic disorder (BDD) guidelines [4] propose that individuals experiencing OCD symptoms are provided with a course of CBT including exposure and response prevention (ERP). The extent of functional impairment, determines whether the initial treatment offered is a low intensity intervention i.e., brief individual CBT including ERP supported by a health technology or self-help manuals requiring less than 10 h of therapist contact, or individual CBT including ERP with more than 10 h of direct therapist contact. Whilst these recommendations are clear, only a small number of people with OCD access a recommended treatment [17]. Furthermore, despite guidelines advocating a low intensity treatment approach, in practice, the majority of patients presenting with OCD symptoms are stepped up to a high intensity treatment, irrespective of their degree of functional impairment.

The effectiveness of low intensity interventions such as computerised CBT or guided self-help for OCD is an emerging area [18–22]. Their incorporation into service delivery models has the ability to encourage better resource efficiency, and thus have potential economic benefits for services, responding to patient needs more promptly.

Whilst it is imperative that services provide evidence-based psychological therapy interventions and improve choice for patients, it is also important that the practitioners delivering the interventions are consulted regarding their views about new treatment options and their delivery methods. Psychological wellbeing practitioners (PWPs) provide support to patients experiencing mild to moderate depression or anxiety using low intensity interventions (interventions involving 10 h or less contact supported by health technologies such as self-help manuals or computers). Their role is considered to be one of a ‘coach’ rather than traditional therapist [23]. Despite having similar therapeutic underlying features these interventions are different to high intensity interventions such as cognitive behaviour therapy (CBT) which require more therapist contact.

Patient acceptability of low intensity interventions with practitioner support has been explored in recent literature [24–26]. However there is limited empirical evidence regarding practitioner experiences and acceptability of supporting patients using these interventions [27, 28]. Despite the successful incorporation of low intensity interventions it has nonetheless encountered some resistance from services and practitioners devoted to the more traditional delivery formats of psychological therapies [29]. The shift towards a low contact, high volume approach [30] has additionally identified the importance of monitoring the effectiveness of the workforce delivering these interventions with evidence suggesting that better patient outcomes are achieved by more efficient practitioners [31]. It is therefore imperative to allow practitioners’ voices to be heard to ensure that they engage with the service delivery model, ‘selling’ it effectively to the patients that they support.

This paper draws upon the experiences of psychological wellbeing practitioners (PWPs) who supported patients as part of a large multi-site randomised controlled trial conducted between 2011 and 2015 [18].

## Methods

### Research context

The trial explored the clinical and cost-effectiveness of two low intensity interventions for adults with OCD – guided self-help and supported cCBT compared with a waiting list for high intensity CBT. Support for both interventions was provided by a PWP over a 12-week period. 473 patients took part. In the short term (3-months) and long-term (12-months) access to guided self-help or supported cCBT prior to accessing high intensity CBT did not demonstrate any significant clinical benefit. However access to the low intensity interventions led to a significant reduction of high intensity CBT uptake and the interventions were better value for money without compromising patient outcomes in the long-term.

*Guided self-help* comprised of a self-help book *‘Overcoming OCD: a workbook’* [32]. Aligned with NICE guidance it was based on CBT principles with a focus on ERP. Weekly guidance was offered face-to-face or telephone, dependent on participant preference. The initial session was 60 min in length with up to 10 additional sessions of 30 min.

*Supported cCBT* comprised of a commercial cCBT program specifically designed for the management of OCD, OCFighter (CCBT Ltd, Birmingham, UK - www.ccbt.co.uk). Participants were given personal access to the online system, whereby their progress could be monitored by their allocated PWP. The program consisted of nine ‘steps’ that incorporated a CBT approach with ERP. They received six, 10 min telephone support calls over the 12-week intervention period. Participants were advised to access the programme as frequently as they would like but six times as a minimum. Lock-out time points of 24 h were imposed automatically after the completion of specific steps to allow for the time for consolidation of learning prior to commencing the next step.

Participants aged 18 and over on a waiting list for high-intensity CBT were recruited from IAPT services in 14 NHS England Trusts. Participants fulfilled the Mini International Neuropsychiatric Interview (M.I.N.I.) [33] criteria for an OCD diagnosis, scoring ≥16 on the self-rated Yale-Brown Obsessive Compulsive Scale (Y-BOCS) [34] indicating a moderate level of OCD. A total of 473 participants took part in the trial.

Two hundred four PWPs from 14 NHS Trusts attended a 3 day training course covering the presentation of OCD and recommended treatments, the trial rationale, design and procedures and supporting patients using the two low intensity trial interventions. PWPs were supported by a clinical trial team member or therapists working within their IAPT service during fortnightly supervision sessions lasting up to 30 min.

### Sampling and recruitment

The study was conducted using a qualitative descriptive design [35] stemming through a post-positivistic lens, taking into account individual constructions of reality. Sampling was purposive, with individuals selected according to their level of trial engagement and involvement of delivering a non-routine intervention in practice. Variance in the characteristics and cultures of the sites in which they worked was additionally taken into account to capture different experiences of supporting patients. Invitations were sent to PWPs who had delivered one or both of the trial interventions to at least one participant. This included PWPs working in IAPT, primary care or secondary care mental health services in the 15 NHS Trusts. It was not possible to send invites to PWPs from two trial sites - one where recruitment had not commenced due to lack of waiting lists and another due to research governance delays.

Recruitment of PWPs took place between October 2013 and March 2014. An invitation letter, information sheet and consent-to-contact form were emailed to all eligible PWPs (*n* = 71). Those who expressed an interest in taking part by returning a completed consent-to-contact form were contacted by a trial researcher. The researcher addressed any queries and arranged a convenient interview date. Participation was voluntary and no reimbursement was offered. Verbal, recorded consent was obtained prior to each interview commencing.

A semi-structured interview schedule was developed by the research team drawing on prior knowledge of the research area and in consultation with a PWP involved in the trial at one of the trial sites. Following piloting with an independent PWP not involved in the trial at their site, minor changes were made. The interview schedule explored:

- personal views of the low intensity treatments they delivered (guided self-help and/or supported cCBT)
- perceived influences on patient engagement and outcomes
- challenges or issues experienced whilst delivering the intervention
- anticipated barriers and facilitators to implementing guided self-help and supported cCBT in routine practice
- views on their involvement in the trial as a whole

A qualified PWP involved in the delivery of the trial interventions was subsequently employed to conduct all of the interviews. The PWP avoided disclosing their personal experiences and background to avoid influencing participants’ responses. Qualitative training was provided by two experienced researchers working on the trial team. All interviews were conducted over the telephone. Interviews ranged in length between 18 and 125 min.

### Analysis

Interviews were audio recorded with participant consent and transcribed verbatim. NVivo qualitative data analysis software [36] was used to assist with the management and analysis of the data.

Four researchers were involved in the analysis of the interview transcripts. Two researchers collaboratively led on data analysis independently coding each transcript while two researchers with different experiential backgrounds independently verified the coding. Personal interpretations of the data and discrepancies were discussed amongst the team and resolved to produce a shared coding manual. Extracts of the coding manual were presented to the wider research team and an independent steering committee of mixed disciplines to ensure that the interpretation was grounded in the original data.

To identify emerging themes in the data, the constant comparative method [37] was used alongside the application of thematic analysis. Unlike grounded theory, where the constant comparative method derives, thematic analysis facilitates constant comparative techniques. Data was systematically compared to inductively identify and affirm emerging patterns, or themes in the data. Analysis of data occurred simultaneously with data collection until saturation of the data was achieved. During analysis the coding manual was modified accordingly to introduce and reshape identified themes. Interviews with practitioners were scheduled to occur after the trials primary outcome point to reduce the possibility that interview participation would contribute to performance bias in their subsequent intervention delivery. Qualitative data were analyses independently and without knowledge of trial outcomes, in order to avoid biasing these analyses, as recommended by the MRC process evaluation model [38].

## Results

### Sample

All PWPs who supported patients in the trial were invited to participate in the qualitative study. Of the 71 invited, 20 responded to the initial invitation (28.2%), all of whom subsequently participated in an interview. Table 1 provides characteristics of the PWPs who took part alongside the characteristics of the PWPs who supported patients in the trial and completed a demographic questionnaire.

**Table 1:** Characteristics of PWPs

Characteristic	Category	Consenting PWPs *n* = 20	All PWPs delivering trial interventions *n* = 68^a^
Age range (years)		24-59	24-61
Mean age (years, SD)		34.1, 9.49	33.9, 10.8
Gender	Female	18 (90)	59 (87)
N (%)	Male	2 (20)	9 (13)
Highest educational qualification N (%)	Undergraduate degree	7 (35)	12 (18)
	Post Graduate Certificate	8 (40)	30 (44)
	Post Graduate Diploma	3 (15)	14 (21)
	Master’s Degree	2 (10)	9 (13)
	PhD	0	1 (2)
Length of time in PWP role N (%)	6mths – 1 year	1 (5)	6 (9)
	1-2 Years	4 (20)	17 (25)
	2 years – 5 years	15 (75)	44 (65)
Received OCD training as part of IAPT training N (%)	Yes	9 (45)	37 (54)
	No	11 (56)	31 (46)

^a^missing data from 3 PWPs

PWPs represented 11 of the 14 Trusts involved in the trial and had a similar gender and age distribution to the overall sample. On average, practitioners had supported 4 patients. Three-quarters (75%) had been in post for more than two years and 45% had received OCD-specific training prior to the trial.

Six main themes were identified, reflecting both patient-centred and professionally-relevant issues that contributed to views on the acceptability of the interventions. Those concerned with patient matters will be presented first followed by those related to the implementation of the interventions and professional beliefs and expectations. Direct quotations will be presented for illustration purposes. ID number, gender (male (M) or female (F)) and length of experience working as a PWP is given (<1 year, 1-2 years, 2-5 years).

### Patient centred issues

#### Flexibility in intervention delivery and fit with patient lifestyle

PWPs reflected upon the theoretical underpinnings of a stepped care approach, highlighting some of its benefits. Maximising the availability of support available to people experiencing OCD symptoms was valued and the accessibility of low intensity interventions as a first-line approach within this model was regarded as a considerable advantage.

*‘I think that it’s made me feel quite hopeful about the intervention being used at step two and the usefulness of a brief intervention rather than the person being on a longer waiting list to be seen at step three. They could get a lot of benefits from the interventions available at step two.’* (167,F, 2-5 years)

In addition to increasing access to psychological interventions in society in general, the ability to enhance experiences on an individual level was acknowledged. PWPs indicated that providing a choice of approach and flexibility was important in order to meet individual needs. The ability to offer a choice of remote or face-to-face support was reported to be of considerable value:

*“We did an initial assessed appointment assessment face to face and then we did the first treatment face to face, and the rest were delivered* via *the phone, and it was also during a late appointment, so it was quarter to six when I contacted the client, which was fantastic for her because otherwise she wouldn’t have been able to attend the treatment because she couldn’t have got off work. She had work commitments, and especially the amount of sessions that we were offering; it would have been really difficult for her to be able to access that treatment if it was nine to five… There were no problems over the telephone at all. The client prefers it. It was more accessible for her, and delivering the treatment was no different to face to face really.*” (89, F, 2-5 years)

Further discourse regarding the supported cCBT intervention drew upon distinctive elements that were considered to be advantageous. The interactive elements of the programme and the ability to revisit sessions were considered to meet patient needs that traditional face-to-face psychological therapies may not. Practitioners highlighted the benefits such as accessibility and anonymity unconstrained by service resources or operating procedures:

“*overall I thought it was a really, really helpful package, and I think if people were able to access that and work on that much more freely…I think it would have a really positive impact on people with OCD symptoms. Especially for people who may be working in the day, or need to be able to access something at a time convenient for them … I guess with accessing an online programme and opting for telephone calls it’s slightly more anonymous, there’s a slightly more private aspect to it, and I think some people prefer that, they want the support and the advice but maybe don’t want to have to meet someone, or see someone. So, I think it would be quite well accepted, once people understand the rationale behind it*.”(09, F, 1-2 years)

### Need to tailor interventions to fit with patient need

PWPs regarded OCD as a complex disorder, with different presenting features to anxiety and depression, conditions they felt experienced in working with. OCD symptoms were considered as being *ingrained*’, requiring ‘*undoing or deep level work*’ and that ‘*it takes a lot to break it [OCD]*’. Therefore, not surprisingly, professionals recalled their initial scepticism about the appropriateness and potential efficacy of low intensity interventions for this group. Negative outlooks for the majority became more positive following receipt of experiential learning via the OCTET trial.

‘…*once [*guided self-help*] is up and running, people have got a really good knowledge of what they need to do, it’s just continuing to implement it and managing with the challenges that are faced as a result of implementing it. But, I think it’s very, very clear and very easy to deliver, and I think that’s what was really good about it, and very fitting to a person’s problem as well, because the compulsions often take over their day, their time. And actually, it’s an intervention that allows a person to gain back some time, and some of their life in terms of engaging in other things as well*.’ (09,F,1-2 years)

The heterogeneous symptom profile of OCD was stressed, and in response the need to tailor any psychological interventions to individual patient needs, preferences and expectations was key. The ‘prescriptiveness’ of the intervention, however, was still valued:

*‘I’d always adapt, and then try and pace it and deal with whatever the patient presents. So it’s prescriptive but flexible. It’s down to you as the therapist to meet the needs of the patient. [The interventions] gave us the steps and so on, so I’d got a clear plan on how to do it and deal with it [OCD], and then it sometimes is that flexibility, when to go back and repeat the early steps and motivate the patient and bits and pieces. So it’s prescriptive, but on the other hand you are using your own knowledge and experience to adapt to the patient’s needs.’ (158,M,2-5 years)*

PWP acceptance of low intensity interventions was driven by the opportunity to offer flexibility in one-to-one support delivery. Weekly sessions were considered by some to be difficult to fit into patients’ lifestyles with some considering more time between sessions would be beneficial, allowing patients more time to consolidate their learning and skill development and putting such skills into practice.

*“…all the documentation says weekly therapy is seen to be more effective. I’m not certain it is, especially if you’re talking in a CBT format, because patients have to learn set techniques and start practicing them, which can often take, I think, longer to understand and interpret the information and nibble away into forming that practice of the techniques and repeating it*.” (158, M, 2-5 years)

Opposing views, however, stressed the importance of more frequent sessions to promote patient engagement and ability to keep on track with their goals:

“*It was just all about consolidating what the treatment was and how the client was putting it into practice, as well as like…she’d say for example…we’d review the week and she’d remember an incident where she maybe didn’t complete the ritual whereas say*, *for example*, *over two weeks she might have forgotten that incident so that consolidation may have been lost a little bit*.” (89, F, 2-5 years)

One PWP identified that their apprehensions about the shorter number and length of support sessions offered for patients accessing OCFighter related more to their own concerns and anxieties, which were challenged over time.

“*I was a bit worried in case that wasn*’*t enough support for the person*, *because I know that the person I*’*d worked with*, *had had OCD for a lot of years*, *and this is where the scepticism came into it. Because I thought*, *I*’*ve only given them 15 min six times*, *and I suppose that didn*’*t seem very much for someone who had had OCD for a long time*…*It was sufficient*, *I think that was more about me. Me wanting to give the best I could*, *in a little amount of time. But I felt a bit mean really*, *I know it sounds a bit daft*, *but I felt a bit mean*, *because I thought I was not giving enough. But*, *it obviously was enough*.” (181, F, 2-5 years)

Ambiguity surrounding the impact of regular support on patient motivation again addressed the need to be patient-centred and also to work within the working aims of a low intensity service:

“…*different people want* [*a*] *different kind of approach*, *so someone wanted to come every week*, *others wanted to leave a bit longer between sessions*…*I couldn*’*t force them to come every week*, *so when it was clear that they wanted a bit longer between sessions we just had to book them in two weeks from that point.*” (93, M, <1 year)

“*I think there's probably a reason that we have step two interventions as four to six sessions … it does keep it contained…I think eight at the most…otherwise if it's not shifting after that then it's step three.*” (111, F, 2-5 years)

### Integration between new treatment models and existing service protocols

Despite their being a positive outlook in supporting patients using a low intensity approach, challenges of implementing into current service delivery models were identified. Resistance from high intensity therapists working within more traditional psychological therapy delivery models was anticipated, with a general recognition that their abilities would be challenged. Identifying the commonality of resistance to change one PWP identified that for implementation to be successful the *‘bigotry’* (43) would need to be overcome by other [team] members:

*“…some of the higher level workers have a distain or a disbelief in PWPs appropriately dealing with it following the OCTET programme.”* (158, M, 2-5 years)

Others reflected upon their own support needs as key to the success of implementation. Drawing upon inconsistencies between services in supporting patients with OCD at step two, standardising training to enhance practitioner competencies and confidence was stressed as vital to the success of implementation of low intensity psychological interventions for OCD. PWP discourse also highlighted the need to maintain organisational standards while applying a consistent approach in managing patients:

*“I think definitely a real need to look at what training the PWPs have for OCD if it was going to be taken forward is really important, because something that I have noticed that actually they're doing well and oh, it all looks good on paper. But actually my concern is yeah, how good is this looking? And I wonder…it's about I think some element of training would be needed to make people who, for example, are just starting out and are newly trained, to get them to look out for that really I guess.”* (111, F, 2-5 years)

One of the particular challenges that participant interviews revealed related to the perceived difficulties implementing the trial intervention delivery protocol within an economic-driven service constrained by the availability of resources. Despite demonstrating the feasibility of implementing low intensity interventions for OCD and delivering acceptable training, challenges to integrating within current service provision models was considered as a potential barrier and concern:

“*Well the length of appointments is very similar, it would be, like, a 45 min assessment and then half an hour follow up or 25 min follow ups, which is very similar to what we do, I suppose it’s the number of sessions which we are a bit more limited at the moment, but that would be really hard I think to convince, kind of, given the present financial climate, I mean, it would need to have good justification to offer more sessions to anyone at this point with any problems. So I think it’s the justification, if it’s there and if we’ve got a good argument why we would need to, kind of, offer a few more sessions to these patients, then it’s possibly something that could happen, it wouldn’t be easy I think, but it’s possible”* (93, M, <1 year)

*“It would be nice if we could offer it, yes. I think the cost implications of the package [OCFighter] of our service probably would make it unlikely we'd be able to offer it.”* (128, F, 2-5 years)

### Limitations in the model used to deliver low intensity interventions

Self-managed therapy approaches, in contrast to more traditional forms of psychological therapy, provide limited direct practitioner support to patients. The role of the PWP in delivering guided self-help and supported cCBT was to guide and monitor patients through the intervention thus influencing the opportunity to engage in therapeutic care. The specificity, structure and resources associated with the interventions were consistently valued as means of successfully supporting patients within a time-limited framework:

*“I think having a very clear package of information, it [trial interventions] clearly defined what to focus on in particular sessions, and it gave a good timeline really of how to work with someone with OCD. And, I think it means that the sessions are very focused, and have a good plan and a good agenda already in place, and I think that would make it more straightforward and take out maybe some of the fear that people might have in working with OCD who aren’t familiar with it*.” (09, F, 1-2 years)

Practitioner’s not only reflected upon their own personal views but considered how the interventions would be perceived by patients. In addition to encouraging engagement, the projection of service credibility via the structured interventions was advocated:

*‘…it helps towards them having…this looks like a professional, serious…is going to work, but…yeah. Even if you didn't show them the book and you worked through and went through it section by section and they never saw the book, I think they'd still benefit. But this adds to the credibility of the delivery of this intervention at this level, and that it's taken seriously and it's being invested in and it's not just a bit of paper.’* (84,F,2-5 years)

Despite the guided self-help intervention being seen as acceptable, in evaluating supported cCBT, specific barriers to patient engagement were identified, predominantly concerning the functionality of the programme. PWPs recognised that these issues went beyond patient usability, impacting on their ability to support patients within the defined protocols. Technical issues were frequently thought to have hampered the progress and engagement of patients and for some PWPs was more time consuming for them than was originally anticipated.

“*I had one guy, he had two laptops and a computer and they didn’t work on any of them. Of course then you ring for the next appointment and say, well, ring [the help desk], sort out the problem, but I always said to them, if you are having any more problems, ring me back, don’t wait for the next appointment. You wouldn’t hear anything, think everything was okay and then they DNA. You’d find it really difficult to get hold of them.”* (130, F, 2-5 years)

The program’s ‘time-lock’ restrictions, which imposed a minimum delay between completion of one step and the start of the next, was also highlighted as a potential barrier to engagement. One participant explained how this reduced his ability to access the supported cCBT:

“*One guy pointed out, if I don’t finish one step until nine o’clock at night, he said it’s not going to be available to me again until nine o’clock the following night. He said it should be 24 h from when he starts the program, because by the time nine o’clock the next night comes round, he said, I’m too tired to go on to it. He’s then got to wait another day to get on to it, so he loses the flow, which I thought was a fair point really*.” (130, F, 2-5 years)

### Capacity to develop confidence and skills

In addition to initial uncertainty about treatments being suitable to meet patient need, there was related concern about whether the disorder was too challenging for the PWP. Lack of initial self-confidence in having the necessary skills and competencies to effectively treat people with OCD, was highlighted by a number of the health professionals interviewed. The majority of PWPS had limited experience in in supporting patients with OCD, partially due to previous guidance recommending that they be referred to higher intensity interventions. Some expressed concerns about their ability to achieve a positive outcome at step two, while others held the view that due to the complexities associated with OCD that low intensity interventions would not be appropriate.

*“I think initially I was very eager to get on board but again there was I guess a bit of anxiety. It’s not something that I was actively treating at Step 2, so I think there was an initial bit of anxiety there about thinking…Is this something we can do at Step 2?…As it was something that we didn’t actively treat, we kind of thought it was more a Step 3 intervention, so I think it was more the lack of knowledge of what actually went on at Step 3 and how that could be used at Step 2 in a sort of a low intensity way.”*(89, F, 2-5 years)

Despite initial concerns of the ability to relieve their own anxieties and meet participant’s needs, PWPs reflected upon the impact the training, received as part of the trial, had upon their confidence. They considered it a vital in relation to developing their knowledge of OCD, development of skills and familiarisation with the intervention materials. Aspects of the training including the opportunity to role play, skills-based practice and the ability to access top-up training when there had been a delay between training and recruitment of participants was valued.

Development of confidence was most apparent in supporting patients using guided self-help, where the availability of written resources was positively regarded and helped to keep patients on track”:

*“I think that the training which we had, it was quite comprehensive…I think it helped with me feeling more confident to deliver it.*” (167, F, 2-5 years)

*“Once I’d overcome that my [lack of] confidence in delivering both [interventions] I was okay, I felt quite confident in delivering both types of support.”* (09, F, 1-2 years)

### Capacity of low intensity interventions to disenfranchise practitioner role

The underlying nature of the PWPs was evident as influencing their acceptability of the two interventions. Their perception of the importance of collaboratively working with patients and developing a positive therapeutic relationship with them to engage and respond to their needs using an individualised approach was considered vital. These deep-rooted beliefs were evident in some of the concerns and difficulties they experienced in performing their role in the trial:

“*Well, it’s part of basic makeup. There’s a rescuer in me and a teacher in me. I enjoy that interaction, and I enjoy having to reinterpret to fit the client’s individual needs. So I have to interpret my knowledge, discover the individual and interpret my knowledge to fit their needs and motivate them to move forward, compared to computer work which is supporting them and trying to motivate them if they’ve got difficulties. I’m still using those skills, but it’s very much redirecting them to the programme and keeping them working on the programme*.” (158, M, 2-5 years)

These challenges were more apparent when supporting patients using supported cCBT in comparison to guided self-help. PWPs felt disengaged with their professional role, expressing that they were ‘absent’ or had taken ‘a back seat’.

*“I don’t think that they took it [supported cCBT support call] quite as seriously because they didn’t really need us if their program was working okay…they had everything there on the computer and for us it was just kind of touching base with them. It wasn’t really going into as much depth as you would with the guided self-help because they were doing it themselves. You felt a bit like you were just technical support if you see what I mean.”* (130, F, 2-5 years)

With the majority of PWPs identifying client contact as a significant part of their role, despite the increasing inclusion of remote delivery models arising within service delivery models, concerns related to this way of working were raised. Some had not has the opportunity to use this approach previously, while others indicated that it was not aligned with their preferences and beliefs. The majority of these views related specifically to the delivery of the supported cCBT intervention where, due to time constraints, it was likely that offering support face-to-face would not be feasible:

*“I just prefer to be sit talking to people because I’ve worked for a telephone service that only delivered telephone interventions for a year and I found it very difficult and as a practitioner I missed seeing people and talking to people and seeing their body language and seeing them feeling better rather than just talking to somebody over the phone*…*I just felt like I was in a call centre rather than being a clinician working with patients who had difficulties*.” (177, F, 2-5 years)

Despite these attitudes, PWPs who had supported patients using both approaches reflected upon their beliefs, and their initial preference for guided self-help. They identified that their beliefs, and perceived role, may be at conflict with the needs of the patients accessing the interventions:

*“…with the cCBT…you have six sessions … ten minutes each…clients really took to that. They didn’t complain about not having enough support whereas the guided self-help it does offer people more support and I feel that people take what they’re given, and I think it made me realise that actually some people are motivated enough, can get on with the programme, and just have very little support from a clinician.*” (205, F, 2-5 years)

## Discussion

This study explored psychological wellbeing practitioner acceptability of supporting people with OCD using low intensity psychological interventions; guided self-help and supported cCBT. Practitioners’ views drew upon salient personal, patient and service-specific issues. Provision of both interventions was regarded positively as a means of helping to overcome existing mental health service delivery barriers, providing people OCD with greater access, choice and flexibility.

OCD was considered by practitioners as a complex condition and, with many having received no prior training to support patients with this condition, many expressed they were initially apprehensive. Their concerns reflected practitioners’ perceived ability to support patients experiencing OCD but also the suitability of a low intensity intervention, most commonly referred to a high intensity service, to meet the needs of this patient group. Initial anxieties about supporting patients that are perceived to be beyond the intended remit of their role are common among mental health staff [27, 28, 39, 40]. It was evident that the provision of training was vital in preparing practitioners to support patients and to allay concerns and change attitudes. Training and supervision are integral to the IAPT model and considered as a playing a key role in its success [41, 42]. Thus if national implementation of these low intensity interventions were to occur the importance of ensuring IAPT codes of practice are adhered to is vital.

Despite identification of the benefits of these interventions under adequate training and supervision conditions, challenges to implementation were evident. Practitioners frequently referred to their self-perceived role and responsibilities and their role as perceived by others. Practitioners identified themselves as distinct to those working in high intensity or psychological services. Referring to guidelines [4], they acknowledged that OCD was not a condition that they usually managed and that resistance was evident from other therapists regarding the suitability for them to do so. Such clinical, and at times political, disputes are evident within mental health with some specifically relation to IAPTs place within the context of mental health delivery [29].

Low intensity interventions are, as with the delivery of most psychological therapies, time-restricted and protocol driven. Practitioners acknowledged significant benefits of this delivery model in terms its ability to fit with patient lifestyle and enhance individual experiences. However apprehensions were evident regarding the extent and method of contact that at times was thought to limit the ability to develop a therapeutic relationship, widely considered as central to patient outcomes. These findings highlight that the shift in mental health care delivery towards more remote working practices to improve efficiency and reduce costs may only exacerbate this problem. Nevertheless it must be acknowledged that the views relating to patient centred issues presented are those of the practitioners involved in this study, and may therefore not reflect precisely or completely those of patients receiving support. Patient experiences of the trial interventions, presented elsewhere, provide further evidence of treatment expectations held by patients, accentuating further the importance of interpersonal aspects of their relationship with the PWP [24]. Consideration of these views, in addition to those of practitioners, is therefore imperative to ensure continued workforce support and engagement.

The importance of the therapist emerged as a central thread throughout the data, with the expectation of many users focusing on the interpersonal aspects as key components of ‘good quality’ care. The presence of a therapeutic relationship is often considered key [43]. Remote working, where non-verbal cues are lost, changes the nature of this relationship and it has been considered as ‘a high risk delivery model’ [39] despite being clinically effective. This may have influenced the finding that guided self-help, which combined the accessibility of self-help materials with support from a professional, was considered an acceptable compromise, consistent with other research.

Research has demonstrated that in incorporating new technology into healthcare delivery models, its success is likely to be determined by the beliefs of its users and its underlying properties [44]. With respect to the individual interventions explored within this study, their credibility was identified as an important factor influencing views on acceptability. Guided self-help was viewed as most successful in its implementation. It was generally perceived to fit with practitioners’ perceived role and was more in-line with current working practices. Despite being seen as an innovative approach to overcoming treatment access, technical difficulties experienced by patients receiving supported cCBT impacted upon practitioners’ ability to support patients, their perceptions about the intervention’s ability to meet patient needs, and raised concerns about its sustainability. High quality resources and good accessibility was viewed as key to service development.

### Limitations

It may be argued that as participants in the study comprised of individuals who had actively participated in the main trial, that the findings may not be generalisable beyond this professional sample. However, as demonstrated previously the study sample was very similar in terms of personal characteristics and experience to the whole trial sample. Additionally, it should be acknowledged that many PWPs who took part in this study were representatives of Trusts where involvement in the trial was mandatory and therefore the sample may not have favoured professionals working within an organisational culture that was motivated to support such interventions.

One researcher conducted all of the interviews, which helped to maintain consistency. It is however recognised that as this individual was also previously involved in supporting patients within the trial, that objectivity may have been compromised. The incorporation of a semi-structured interview schedule and independent data coding by four researchers, one of whom was a PWP with no previous experience of the trial minimised the impact of this. The researcher did not disclose their professional background to any of the participants. This was an intentional decision to help encourage participants to elaborate on their experiences, rather than to assume prior knowledge on the part of the researcher. It is recognised that their experience and understanding of service delivery and trial procedures may have enhanced exploration of significant issues. Conversely however, admission of professional role may have encouraged trust and openness on the part of participants who may have felt able to relate to the researcher more closely.

Also to note is that all interviews were conducted by telephone. As with therapeutic relationships, recognised previously, although the impact this may have is unknown [45] the rapport between the interviewer and participant may have been affected by the inability to recognise and respond to non-verbal cues. However, the PWP conducting the interviews was experienced in delivering support via this modality and in overcoming some of the challenges that the inability to observe non-verbal behaviours can bring. The opportunity to take part by telephone is perceived to have facilitated recruitment with a population that can often be hard to engage given their significant workloads.

It is recognised that the practitioners who took part had accumulated, on average, more years of professional experience working as a PWP than the overall trial sample. Consequently it may mean that the views are not reflective of less experienced practitioners working within IAPT services. However, given that less than half of those interviewed had received OCD-specific training prior to taking part in the trial observations relating to self-confidence and implementation into routine services may be reflective of the views of practitioners working within these services.

## Conclusions

Exploring practitioner views within a large research trial provided the opportunity to obtain beneficial feedback regarding the implementation, acceptability and sustainability of new low intensity treatment options for OCD. Practitioners recognised the potential benefits offered by these interventions both from a patient and service delivery perspective, but simultaneously identified potential challenges that may be faced should they be implemented into routine practice. Training and support for practitioners was considered key to ensuring the successful delivery of the interventions and adequate support of patients.

## Abbreviations

BDD: Body dysmorphic disorder
CBT: Cognitive behaviour therapy
cCBT: Computerised cognitive behaviour therapy
ERP: Exposure and response prevention
IAPT: Improving access to psychological therapy
M.I.N.I: Mini International neuropsychiatric interview
NICE: National Institute of Health and Care Excellence
OCD: Obsessive-compulsive disorder
PWP: Psychological wellbeing practitioner
WHO: World Health Organisation
Y-BOCS: Yale-brown obsessive compulsive scale
